# Effective Noninvasive Zygosity Determination by Maternal Plasma Target Region Sequencing

**DOI:** 10.1371/journal.pone.0065050

**Published:** 2013-06-10

**Authors:** Jing Zheng, Chenming Xu, Jing Guo, Yuan Wei, Huijuan Ge, Xuchao Li, Chunlei Zhang, Haojun Jiang, Ling Pan, Weiping Tang, Weiwei Xie, Hongyun Zhang, Yangyu Zhao, Fuman Jiang, Shengpei Chen, Wei Wang, Xun Xu, Fang Chen, Hefeng Huang, Hui Jiang

**Affiliations:** 1 Science and Technology, BGI-Shenzhen, Shenzhen, China; 2 Key Laboratory of Reproductive Genetics, Zhejiang University, Hangzhou, China; 3 Shenzhen Municipal Key Laboratory of Genome Sciences, Shenzhen, China; 4 Department of Biomedical Engineering, Southeast University, Nanjing, China; 5 Department of Gynaecology and Obstetrics, Peking University Third Hospital, Beijing, China; 6 Shenzhen Municipal Key Laboratory of Birth Defects Screening and Engineering, Shenzhen, China; 7 East China Marketing Department, BGI-Shenzhen, Shenzhen, China; 8 Shenzhen Clinical Laboratory, Shenzhen, China; 9 State Key Laboratory of Bioelectronics, School of Biological Science and Medical Engineering, Southeast University, Nanjing, China; 10 Department of Biology, University of Copenhagen, Copenhagen, Denmark; 11 Guangdong Provincial Key Laboratory of Human Diseases Genome, Guangdong, China; VU University Medical Center, The Netherlands

## Abstract

**Background:**

Currently very few noninvasive molecular genetic approaches are available to determine zygosity for twin pregnancies in clinical laboratories. This study aimed to develop a novel method to determine zygosity by using maternal plasma target region sequencing.

**Methods:**

We constructed a statistic model to calculate the possibility of each zygosity type using likelihood ratios (***L_i_***) and empirical dynamic thresholds targeting at 4,524 single nucleotide polymorphisms (SNPs) loci on 22 autosomes. Then two dizygotic (DZ) twin pregnancies,two monozygotic (MZ) twin pregnancies and two singletons were recruited to evaluate the performance of our novel method. Finally we estimated the sensitivity and specificity of the model *in silico* under different cell-free fetal DNA (cff-DNA) concentration and sequence depth.

**Results/Conclusions:**

We obtained 8.90 Gbp sequencing data on average for six clinical samples. Two samples were classified as DZ with ***L*** values of 1.891 and 1.554, higher than the dynamic DZ cut-off values of 1.162 and 1.172, respectively. Another two samples were judged as MZ with 0.763 and 0.784 of ***L*** values, lower than the MZ cut-off values of 0.903 and 0.918. And the rest two singleton samples were regarded as MZ twins, with ***L*** values of 0.639 and 0.757, lower than the MZ cut-off values of 0.921 and 0.799. *In silico*, the estimated sensitivity of our noninvasive zygosity determination was 99.90% under 10% total cff-DNA concentration with 2 Gbp sequence data. As the cff-DNA concentration increased to 15%, the specificity was as high as 97% with 3.50 Gbp sequence data, much higher than 80% with 10% cff-DNA concentration.

**Significance:**

This study presents the feasibility to noninvasively determine zygosity of twin pregnancy using target region sequencing, and illustrates the sensitivity and specificity under various detecting condition. Our method can act as an alternative approach for zygosity determination of twin pregnancies in clinical practice.

## Introduction

It was reported that fetal mortality rate at 20 weeks of gestation or more was 6.22 deaths per 1,000 in United States, in which the fetal mortality rate for twins was 2.7 times higher compared to singletons [1]. The higher risk of twin pregnancies may due to several reasons, for instance, twin–twin transfusion syndrome (TTTS) [2]. There are more than 4,500 TTTS cases per year in the U.S. [3]. Moreover, a significantly increasing risk has been observed in monozygotic (MZ) twins in previous studies [4]. Therefore, zygosity is an important parameter in prenatal diagnosis for twin pregnancies.

The diagnosis of zygosity for twin pregnancies relies on the determination of chorionicity by ultrasound scanning within 14 gestational weeks, with 89.8% sensitivity and 99.5% specificity [5]–[7]. However, the accuracy of ultrasound detection declines dramatically due to thinner chorionicity in the second trimester [8]. Invasive approaches such as amniocentesis or cord blood sampling combined with microsatellite DNA markers could also detect zygosity with high accuracies, but it presents a potential miscarriage at a risk of 0.5–1% [9]. Thus there is a huge demand for a noninvasive method to accurately determine the zygosity type without the limitation of gestational age. The discovery of cell-free fetal DNA (cff-DNA) in maternal plasma opened a new direction for noninvasive prenatal diagnosis [10]. Combined with the rapidly developing massively parallel sequencing(MPS) technology, Qu *et al.* recently observed the fluctuation of cff-DNA concentration among autosomes between dizygotic (DZ) and MZ twin pregnancies. The SD variation of the fluctuation from 8 samples was regarded as the indication to determine the zygosity [11]. However, the method lacked evaluation of sensitivity and specificity.

Herein, we developed a noninvasive method based on maternal plasma target region sequencing to determine zygosity of twin pregnancies. We successfully determined two DZ, MZ twin pregnancies and two simulated MZ twin pregnancies through our mathematical model and obtained satisfactory sensitivity and specificity *in silico*. Our study provides a practical alternative approach for zygosity determination in clinical practice.

## Results

### Bioinformatic Pipeline Establishment

In order to determine the zygosity, we employed a bioinformatic method using a conditional probability model. We defined ***L_i_*** to measure the zygosity tendency of each available paternal-only heterozygous SNP locus (where maternal genotype was homozygous), and ***L*** value which was the geometric mean of ***L_i_*** to represent the global tendency. The zygosity could be determined if its ***L*** value passed its corresponding cut-off.

In order to get the cut-offs, we generated simulated samples with different gradients of cff-DNA concentration from 10.00% to 30.00% and sequence depth from 300× to 1300×, and got a series of real cut-offs (***L_R_***) with the boundaries of >95% confidence interval (CI) (**Table S1**). Based on these scattered ***L_R_***, we used least squared method (LSM) to obtain two approximate mathematical expressions of DZ and MZ dynamic cut-offs respectively (**Materials and Methods**).

After getting the fitting expressions, we established a comprehensive pipeline, which included sequence reads alignment, parental genotype detection, total cff-DNA concentration estimation, calculation of ***L*** among clinical samples and zygosity determination by comparing ***L*** to its corresponding dynamic cut-off. Six clinical samples were recruited to assess the accuracy of our methodology. Finally, we used more simulated samples to depict the sensitivity and specificity of our methodology under various detecting conditions *in silico*.

### Clinical Samples and Data Productions

Four twin pregnancies named Sample1, 2, 5 and 6 were enrolled from Women’s Hospital School of Medicine Zhejiang University and Peking University Third Hospital, whose gestational ages were 20^+2^ and 19^+4^, 20 and 20^+4^ weeks, respectively. We also enrolled two singletons named Sample3 and Sample4 with gestational age of 19 and 8 weeks from Women’s Hospital School of Medicine Zhejiang University and BGI-Shenzhen. Sample1 and Sample2 had already been diagnosed as DZ by invasive procedure aminocyte karyotyping suggesting mixed-gender twin pregnancies. Sample5 and Sample6 were diagnosed as MZ by ultrasound scanning.

4.43 Gbp and 11.47 Gbp clean data were extracted from maternal plasma Sample1 and Sample2, corresponding with 930.87× and 1363.25×sequence depth. 95.81% and 97.51% of target region was covered by at least one read. For maternal plasma Sample3 and Sample4, 4.53 Gbp and 2.55 Gbp clean data were extracted respectively. The sequence depth was 519.34× and 446.89×, and the corresponding coverage of target region was 95.36% and 96.88%. For Sample5 and Sample6, we obtained 16.26 Gbp and 15.59 Gbp clean data, corresponding to 492.3× and 271.2× sequence depth, 99.85% and 98.68% of target region depth (**Table 1**).

**Table 1:** Data production of 6 clinical samples.

Sample	Production(Gbp)	Coverage(%)*	Depth(×)*
Father Sample1	0.41	98.18	219.63
Mother Sample1	0.37	96.01	192.54
Father Sample2	0.46	98.22	228.93
Mother Sample2	0.51	96.29	268.76
Plasma Sample1	4.43	95.81	930.87
Plasma Sample2	11.47	97.51	1363.25
Father Sample3	0.33	95.06	130.30
Mother Sample3	0.38	95.37	146.03
Father Sample4	0.10	92.59	51.47
Mother Sample4	0.37	94.18	185.33
Plasma Sample3	3.16	95.36	519.34
Plasma Sample4	2.55	96.88	446.89
Father Sample5	2.05	99.64	97.36
Mother Sample5	1.51	98.12	76.80
Father Sample6	2.25	99.60	106.58
Mother Sample6	1.26	97.98	64.27
Plasma Sample5	16.26	99.85	492.30
Plasma Sample6	15.59	98.68	271.20

* “Coverage (%)” and “Depth (×)” mean the coverage and average sequencing depth in the target region.

### Estimation of Total cff-DNA Concentration and Zygosity Determination

Genotypes of parental genomes were analyzed by SOAPsnp [12], and only parental-specific homozygous loci in the form of ♀AA♂BB were selected. Then the sequence reads from those loci were used to estimate the total cff-DNA concentration. We obtained 1,209, 1,057, 1,090 and 986, 1,150 and 1,241 parental-specific homozygous loci from Sample1-6 respectively. And the total cff-DNA concentrations of Sample1-6 were estimated at 27.04%, 22.12%, 23.35%, 9.36%, 18.83% and 25.16%, respectively.

**Figure 1:**
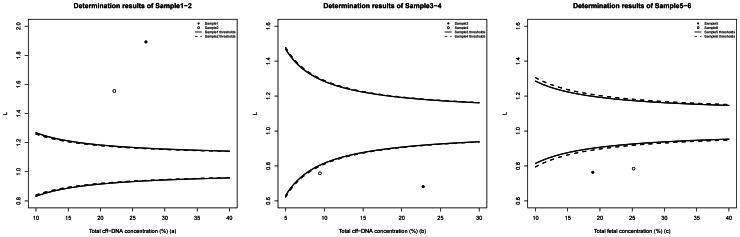
Zygosity determination results of 6 clinical samples. In Figure 1a, the two points were both above the corresponding DZ cut-off, indicating both samples were correctly determined as DZ. In Figure 1b and 1c, the four points were all under the corresponding MZ cut-off, meaning simulated and real MZ twin pregnancies were all correctly determined.

According to our mathematical model, paternal-only heterozygous loci in the form of ♀AA♂AB were used to calculate ***L*** values. 708 and 603 loci were available for Sample1 and Sample2 to obtain 1.891 and 1.554 of ***L*** values, which were both above their corresponding DZ cut-offs (>1.162 and >1.172 for DZ, while <0.938 and <0.928 for MZ), indicating both samples were DZ (**Figure 1a**). ***L*** values of Sample3 and Sample4 were calculated as 0.639 and 0.757 through 564 and 610 available loci respectively, which were both lower than the MZ respective cut-offs (<0.921 and <0.799 for MZ, while >1.179 and >1.301 for DZ)(**Figure 1b**). Additionally, by using 554 and 558 available loci of Sample5 and Sample6 respectively, ***L*** of this two samples were 0.763 and 0.784, both below their MZ cut-offs (<0.903 and <0.918 for MZ, while >1.197 and >1.182 for DZ) **(** **Figure 1c** **)**.The results for these six samples showed the zygosity of twin pregnancies could be determined using our bioinformatic method through maternal plasma target region sequencing.

### Estimation of Sensitivity and Specificity *in Silico*

**Figure 2:**
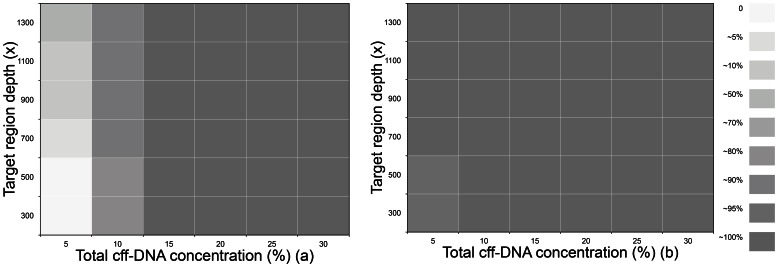
The estimated sensitivity and specificity. Figure 2a and 2b indicated the specificity (DZ) and sensitivity (MZ) with different total cff-DNA concentration and sequence depth respectively. The detailed grey level of each square represented the related accuracy according to the legend on the right, ranging from 0% to 100%.

To further understand the performance of our method, we simulated sequence data with different gradients of cff-DNA concentration and sequence depth (**Materials and Methods**). Overall, the sensitivity, which was defined as MZ accuracy, increased with the enhancement of cff-DNA concentration and sequence depth. It could achieve 99.90% on the condition of 10.00% total cff-DNA concentration and 300× target region sequence depth (**Figure 2a**). Also, the specificity (DZ accuracy) of 15.00% total cff-DNA concentration and 500× target region depth was as high as 97.00% (**Figure 2b**). It was notable that the results from *in silico* showed a relatively high accuracy to determine MZ twins than DZ twins, which might be partially related to the systematic loss of paternal-specific alleles in the maternal plasma sequence data. Meanwhile, we also found that the total cff-DNA concentration plays a more decisive effect than the sequence depth in the zygosity determination. (**Table S2**).

## Discussion

In this study, we developed a practical method to noninvasively determine the zygosity of twin pregnancies by using target region sequencing for maternal plasma. The method consists of determination of empirical dynamic thresholds, cff-DNA concentration estimation and likelihood ratios calculation. The zygosity types of 4 clinical twin pregnancies samples were determined successfully as well as those of 2 singletons. The simulation data also showed that more than 99.90% of the MZ simulated samples with total cff-DNA concentration as much as 10.00% were correctly determined by using about 2.00 Gbp sequence data. Moreover, the sensitivity was improved apparently with the increment of cff-DNA concentration.

Parental genotypes were necessary information in our current method. Since the judgments of fetal genotypes mostly relied on the prior probabilities ensured by parental genotypes in the Bayesian model, parental genomes helped filtering useless and disruptive SNP loci, e.g. loci in the form of ♂AA♀AA and ♂AB♀AB. Therefore we could use only the paternal-specific heterozygous loci (♂AB♀AA) to calculate the likelihood ratio and estimate the percentage cff-DNA through parental-specific homozygous loci (♂AA♀BB).

Comparing with conventional approaches, this sequencing-based approach has several advantages. Firstly, cff-DNA detection has been reported to as early as four weeks [13], which has much less limitation of sampling time restriction than ultrasound scanning. Secondly, maternal blood sampling avoids the risk of miscarriage carried by invasive procedure. Lastly, we used an empirical dynamic threshold for DZ and MZ twin pregnancies to improve the accuracy of zygosity detection, which could significantly minimize the fluctuation of cff-DNA between different loci.

However, our bioinformatic model still needs to be improved in the following studies. Firstly, we constructed this model on the hypothesis of the same contribution to cff-DNA concentration in twin pregnancies, but previous studies have reported a variable combination of cff-DNA concentration for each fetus. False signal might be obtained in MZ detection if there is a significant bias in the distribution of cff-DNA concentration. Referring to some clinical information such as crown-rump length (CRL), the fractional cff-DNA concentration of MZ twins may be preliminarily ensured.

Secondly, this high throughput sequencing approach could be only used to distinguish MZ and DZ twin pregnancies. The detailed physiological structure of the placenta for MZ twins, such as monochorionic-monoamniotic (MCMA) twins and monochorionic-diamniotic (MCDA) twins, could be determined by only the combination of ultrasound scanning with sequencing test.

Besides ultrasound scanning and invasive prenatal test, few prenatal detection approaches could be provided for twin pregnancies due to limited accuracy. Here we demonstrated a sequencing-based noninvasively approach to detect zygosity, which could give clues for twins specific diseases, such as TTTS, as well as gender determination and sex-linked monogenetic diseases [14], [15]. Our study also encourages the application of sequencing technology using maternal plasma to meet rigorous clinical needs, especially on twin pregnancies.

## Materials and Methods

### Sample Recruitment and Library Construction

Six pregnant women, including four of which with twin pregnancies and the rest two with singleton pregnancies, were recruited for this study. Written informed content was obtained from each participant and approval was obtained from the Institutional Review Board of BGI-Shenzhen. 5 ml maternal blood was drawn into EDTA-anticoagulated tubes, and plasma samples were isolated using two-steps centrifugation. Cell-free DNA was extracted from 600 µl maternal plasma following the instruction of QIAamp DNeasy Blood & Tissue Kit (Qiagen). DNA libraries were prepared in accordance with previous study [16], [17]. We also collected 5 ml paternal peripheral blood to construct the model. Genomic DNA (gDNA) for whole blood were extracted and used to construct pre-capture libraries with 200 bp insert size.

### Targets Regions Capture and Sequencing

We designed two versions of probes, both covering 4,524 SNPs from 22 autosome chromosomes (**Table S3**). The SNPs were selected from dbSNP build 131 with at least of 0.3 of MAF. DNA libraries were hybridized with the capture probes at 65°C for 22–24 hours, in accordance with the manufacturer’s instructions. After hybridization, the captured targets were selected by pulling down the biotinylated probe/target hybrids with M-280 streptavidin Dynabeads (Invitrogen). Then, the targeted-DNA libraries were enriched by PCR amplification. And the PCR products were purified by QIAquick PCR Purification Kit. These libraries were subjected to target enrichment and then precede paired-end (PE) 90 cycles sequencing on Illumina HiSeq2000 Analyzers (following the manufacturer’s standard cluster generation and sequencing protocols). The PE reads were mapped to the human reference genome (Hg19, Build37.3) using SOAP2 [18] with maximally five mismatches. PCR duplication and non-unique alignments reads were also removed before following analysis. The genotypes of 4,524 SNPs for parents and fetus were detected using SOAPsnp. All the raw sequencing data had submitted to NCBI SRA (http://www.ncbi.nlm.nih.gov/sra) and the Submission ID is SRA071774.

### Bioinformatic Model for Zygosity Determination

To noninvasively determine zygosity using maternal plasma sequencing, we constructed a comprehensive bioinformatic model based on paternal-specific heterozygous SNP loci. Those loci provide applicable information to determine the zygosity in the massive background of the maternal homozygotes on these SNPs. We defined ***L_i_*** as a likelihood ratio to measure the tendency of zygosity of a single locus. Through the simulation of 10,000 loci with the same fetal genotype (Type I) and 10,000 with different fetal genotypes (Type II), we discovered the natural logarithm of ***L_i_*** (ln ***L_i_***) of most loci of Type I was lower than 0 while ln ***L_i_*** of most loci of Type II was higher than 0 (**Figure S1**). As most loci could increase the signal-noise ratio in our zygosity determination, we used the cumulative difference ***L*** brought by all ***L_i_*** to enrich the signal and regarded it as the effective evidence to determine the zygosity.

We firstly calculated cff-DNA concentration based on parental-specific homozygous SNP loci. For each available biparental homozygous SNP locus (♂AA♀BB), where the fetal genotypes of both twins are definite to be AB, we calculated the ratio 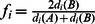 as the percentage cff-DNA from this locus, where ***d*** meant the depth of the allele A or B. Then the percentage cff-DNA was estimated by calculating the average value of all the ratios. The total calculating formula is:
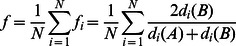



As for the detailed calculation, for each available paternal-specific heterozygous SNP locus (♂AB♀AA), the conditional probability of DZ twins was calculated as:
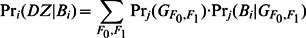



Conditional probability of MZ twins was calculated as:
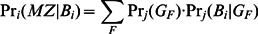



In the equation, ***F_0_*** and ***F_1_*** stood for DZ fetuses and ***F*** for MZ fetuses; ***G_F0, F1_*** and ***G_F_*** mean genotype for fetuses; ***B_i_*** mean the observation of base distribution at a typical locus in maternal plasma; ***j*** stood for the number of fetal genotypes.

Theoretically, genotypes in paternal-specific heterozygous loci should be the same in MZ twins, while probably different in DZ twins. Here we used ***L_i_*** as an odd ratio between the conditional probability of DZ and MZ twins pregnancies to quantify the tendency of zygosity:
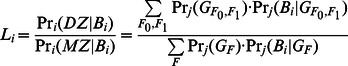



***L_i_*** value should be larger than 1 if there was a DZ twins pregnancies. We employed ***L*** as the numerically cumulative difference of ***L_i_*** to describe the global tendency of zygosity. The total likelihood ratio ***L*** value would be calculated by at least hundreds of paternal-specific heterozygous loci as a geometrical mean:
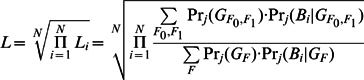



Considering the fluctuation of sequencing depths and cff-DNA concentration, we set a dynamic threshold for ***L*** values to determine zygosity. Of 4,524 autosomal SNP loci, we randomly generated maternal plasma sequence results of 500 paternal-specific heterozygous loci for DZ and MZ twin pregnancies to calculate the likelihood ratio ***L*** value. In order to obtain a series of real cut-offs (***L_R_***) as the boundaries of >95% CI, we simulated six different sequence depths from 300× with 200× of gradient increasing and five different cff-DNA concentrations from 10.00% with 5.00% of gradient increasing, for 500 DZ and 500 MZ samples.

Hereinto we used least squared method (LSM) to get two approximate mathematical expressions of DZ and MZ dynamic cut-offs respectively by using ***L_R_***. It was expressed as:
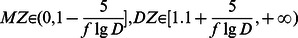
where ***f*** means cff-DNA concentration and ***D*** represents sequence depth. The reliability of the fitting expressions was validated by calculating the multiple correlation coefficients ***R^2^***. The results for DZ and MZ expressions were 0.98 and 0.95 respectively, indicating the reasonability of the expressions. For better understanding of our methodology, we illustrated a three-dimensional figure through our fitting expressions of ***L*** to exhibit the broader feasible region with the enhancement of percentage cff-DNA and sequence depth (**Figure S2**). The figure showed that DZ twins’ feasible region was upon the upper surface, while MZ twins’ feasible region was below the inferior surface. Lastly, additional 1,000 DZ and 1,000 MZ simulated samples were generated to estimate the sensitivity and specificity for different cff-DNA concentration and sequence depth *in silico*.

## Supporting Information

Figure S1 ***L_i_* distribution of two types of loci.** 10,000 loci of Type I, which were represented by using red pillars, meant those with fetal genotypes in concordance. While 10,000 loci of Type II, which were represented by using green pillars, meant those without fetal genotypes in concordance.
(TIF)

Figure S2 **The three-dimensional feasible region of zygosity determination.** The zone beyond the surface above meant the feasible region for DZ twins, while the zone under the surface below meant the feasible region for MZ twins.
(TIF)

Table S1 **Real cut-offs with different total cff-DNA concentration and sequence depth.**
(DOC)

Table S2 **Evaluation results of sensitivity and specificity *in silico*.**
(DOC)

Table S3 **Information about the 4524 autosomal SNP loci.**
(XLS)
