# Supplementary figures and images for: Effective Noninvasive Zygosity Determination by Maternal Plasma Target Region Sequencing
Source: PLoS One. 2013 Jun 10;8(6):e65050. doi: 10.1371/journal.pone.0065050 (PMC3677919; doi:10.1371/journal.pone.0065050)

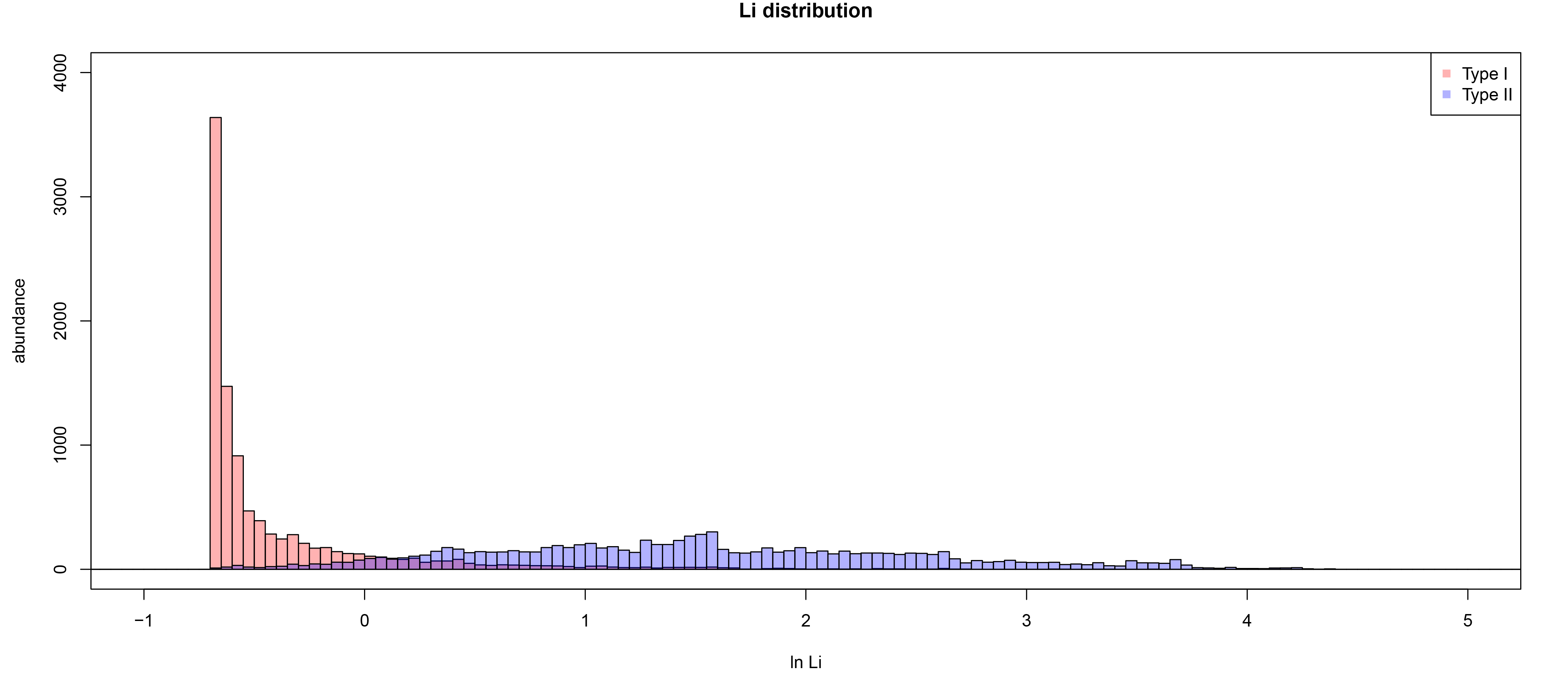

Supplement: Figure S1 — Li distribution of two types of loci. 10,000 loci of Type I, which were represented by using red pillars, meant those with fetal genotypes in concordance. While 10,000 loci of Type II, which were represented by using green pillars, meant those without fetal genotypes in concordance. (TIF) [file pone.0065050.s001.tif]

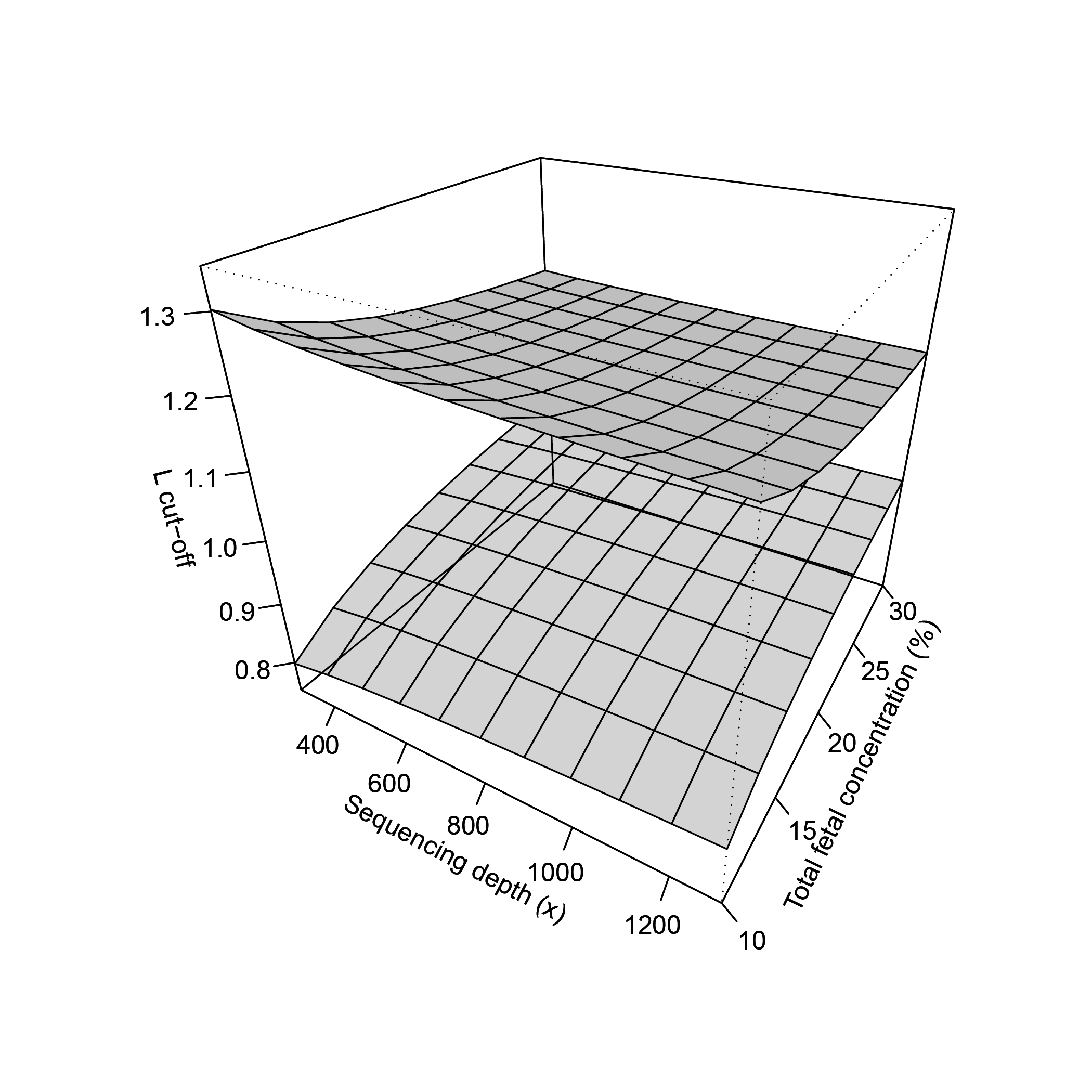

Supplement: Figure S2 — The three-dimensional feasible region of zygosity determination. The zone beyond the surface above meant the feasible region for DZ twins, while the zone under the surface below meant the feasible region for MZ twins. (TIF) [file pone.0065050.s002.tif]
