# Supplementary material for: Effective Noninvasive Zygosity Determination by Maternal Plasma Target Region Sequencing
Source: PLoS One. 2013 Jun 10;8(6):e65050. doi: 10.1371/journal.pone.0065050 (PMC3677919; doi:10.1371/journal.pone.0065050)
Supplement: Table S1 — Real cut-offs with different total cff-DNA concentration and sequence depth. (DOC) [file pone.0065050.s003.doc]

Table S1. Real cut-offs with different total cff-DNA concentration and sequence depth.

| ***f***  **D *L_R_*** | | 10% | 15% | 20% | 25% | 30% |
| --- | --- | --- | --- | --- | --- | --- |
| DZ | 300 | 1.274 | 1.227 | 1.199 | 1.161 | 1.140 |
|  | 500 | 1.264 | 1.218 | 1.196 | 1.161 | 1.139 |
|  | 700 | 1.259 | 1.212 | 1.194 | 1.160 | 1.138 |
|  | 900 | 1.258 | 1.207 | 1.189 | 1.160 | 1.137 |
|  | 1,100 | 1.254 | 1.204 | 1.189 | 1.157 | 1.136 |
|  | 1,300 | 1.253 | 1.201 | 1.188 | 1.155 | 1.130 |
| MZ | 300 | 0.809 | 0.881 | 0.910 | 0.920 | 0.948 |
|  | 500 | 0.810 | 0.883 | 0.913 | 0.928 | 0.950 |
|  | 700 | 0.813 | 0.885 | 0.917 | 0.935 | 0.958 |
|  | 900 | 0.815 | 0.888 | 0.923 | 0.939 | 0.963 |
|  | 1,100 | 0.820 | 0.894 | 0.929 | 0.941 | 0.965 |
|  | 1,300 | 0.834 | 0.908 | 0.931 | 0.942 | 0.977 |

***f*** means the total cff-DNA concentration. **D** stands for the sequence depth. ***L_R_*** represents the values were the real boundaries of >95% CI *in silico.*
