# Supplementary material for: Effective Noninvasive Zygosity Determination by Maternal Plasma Target Region Sequencing
Source: PLoS One. 2013 Jun 10;8(6):e65050. doi: 10.1371/journal.pone.0065050 (PMC3677919; doi:10.1371/journal.pone.0065050)
Supplement: Table S2 — Evaluation results of sensitivity and specificity in silico. (DOC) [file pone.0065050.s004.doc]

Table S2. Evaluation results of sensitivity and specificity *in silico*.

| ***f***  ***D*** | | 5% | 10% | 15% | 20% | 25% | 30% |
| --- | --- | --- | --- | --- | --- | --- | --- |
| DZ | 300 | 0.00% | 78.10% | 95.30% | 100.00% | 100.00% | 100.00% |
|  | 500 | 0.00% | 80.00% | 97.00% | 100.00% | 100.00% | 100.00% |
|  | 700 | 4.20% | 82.00% | 97.20% | 100.00% | 100.00% | 100.00% |
|  | 900 | 7.50% | 83.10% | 97.50% | 100.00% | 100.00% | 100.00% |
|  | 1,100 | 9.30% | 84.20% | 98.40% | 100.00% | 100.00% | 100.00% |
|  | 1,300 | 10.20% | 85.60% | 100.00% | 100.00% | 100.00% | 100.00% |
| MZ | 300 | 92.50% | 99.90% | 100.00% | 100.00% | 100.00% | 100.00% |
|  | 500 | 94.00% | 100.00% | 100.00% | 100.00% | 100.00% | 100.00% |
|  | 700 | 95.60% | 100.00% | 100.00% | 100.00% | 100.00% | 100.00% |
|  | 900 | 96.40% | 100.00% | 100.00% | 100.00% | 100.00% | 100.00% |
|  | 1,100 | 96.90% | 100.00% | 100.00% | 100.00% | 100.00% | 100.00% |
|  | 1,300 | 97.60% | 100.00% | 100.00% | 100.00% | 100.00% | 100.00% |

***f*** means the total cff-DNA concentration. **D** stands for the sequence depth(×).
